# Effect of Segment Types on Characterization of Soft Sensing Textile Actuators for Soft Wearable Robots

**DOI:** 10.3390/biomimetics7040249

**Published:** 2022-12-19

**Authors:** Ayse Feyza Yilmaz, Fidan Khalilbayli, Kadir Ozlem, Hend M. Elmoughni, Fatma Kalaoglu, Asli Tuncay Atalay, Gökhan Ince, Ozgur Atalay

**Affiliations:** 1 Faculty of Textile Technologies and Design, Textile Engineering Department, Istanbul Technical University, Istanbul 34437, Turkey; 2 Faculty of Computer and Informatics Engineering, Computer Engineering Department, Istanbul Technical University, Istanbul 34469, Turkey; 3 Faculty of Technology, Textile Engineering Department, Marmara University, Istanbul 34854, Turkey

**Keywords:** soft robotic, soft actuator, sensing actuator, textile-based actuator, wearable robot

## Abstract

The use of textiles in soft robotics is gaining popularity because of the advantages textiles offer over other materials in terms of weight, conformability, and ease of manufacture. The purpose of this research is to examine the stitching process used to construct fabric-based pneumatic bending actuators as well as the effect of segment types on the actuators’ properties when used in soft robotic glove applications. To impart bending motion to actuators, two techniques have been used: asymmetry between weave and weft knit fabric layers and mechanical anisotropy between these two textiles. The impacts of various segment types on the actuators’ grip force and bending angle were investigated further. According to experiments, segmenting the actuator with a sewing technique increases the bending angle. It was discovered that actuators with high anisotropy differences in their fabric combinations have high gripping forces. Textile-based capacitive strain sensors are also added to selected segmented actuator types, which possess desirable properties such as increased grip force, increased bending angle, and reduced radial expansion. The sensors were used to demonstrate the controllability of a soft robotic glove using a closed-loop system. Finally, we demonstrated that actuators integrated into a soft wearable glove are capable of grasping a variety of items and performing various grasp types.

## 1. Introduction

Recent years have seen an increase in study of soft robotics, with the goal of ensuring the safe interaction of robot technologies with humans and the environment [1,2]. Textile materials are favored in these investigations due to their light weight, softness, conformability to various shapes, and comfort properties [2,3,4,5]. As a result, terminology such as “robotic textiles”, “wearable robots”, and “textile-based robots” began to appear in the literature, and textiles became a component of new generation robots. Naturally passive textiles gain the ability to activate in a number of ways and become dynamic. Textile actuators can be powered by cable-driven (or tendon-driven) systems [6,7,8,9,10,11,12], hydraulic or pneumatic fluid systems [13,14,15,16,17,18,19,20], or shape-memory polymers [21,22,23]. However, fluid-driven actuation is one of the most prevalent and rapidly growing types of actuation in the soft robotics field for developing assistive devices [24]. To create fabric-based fluidic actuators capable of applying forces and defining motions, layers of materials with opposing mechanical properties are joined to form anisotropic inflated pouches. Technologies such as ultrasonic welding [25], thermal welding [5,19,26], and overlock sewing [16] have been used to construct anisotropic actuator shells by combining fabric layers with different mechanical properties. Soft actuator shells are then inflated with air fluid to produce the necessary force and motion. The creation of mechanical anisotropy between fabric layers is essential to obtaining the bending motion of the pressurized fabric actuator [16]. However, a thorough investigation into how the high or low mechanical anisotropy differential of the fabric layers that make up the actuator might affect the actuator’s force characterization is still lacking.

Prior experiments employing silicon actuators have indicated that segmented actuators are advantageous for high energy consumption, low energy density, and delayed response time problems [18,27,28,29,30]. The main goal of this research is to determine how different segment types created by sewing affect the characteristics of fabric-based actuators. Therefore, we explored how different segment types affected the actuators’ grasping forces and bending angles. To accomplish this, we first combined commercially available woven and weft knit fabrics with an asymmetry and anisotropy approach, followed by demonstrating the design of the soft bending actuator using sewing technology. We evaluated the influence of varying anisotropies between layers using two distinct knit and woven textiles. To show the actuators’ suitability for wearable applications, they were employed to make a soft robotic glove. With the expansion of soft robotics into more complex and demanding applications, sensor data become critical for obtaining high task performance levels. Thus, we combined our previously textile-based capacitive sensors [31] into optimally characterized actuators and showed their use in a wearable exoskeleton glove using a closed-loop system design.

## 2. Materials and Methods

### 2.1. Fabric Materials

Two woven and two weft knit textile fabrics with varying structure properties were used in the experiments. Table 1 lists the properties of knit fabrics that are coded as K1 and K2, while Table 2 lists the properties of woven fabrics that are coded as W1 and W2.

### 2.2. Fabrication of Soft Fabric Actuator

**Figure 1:**
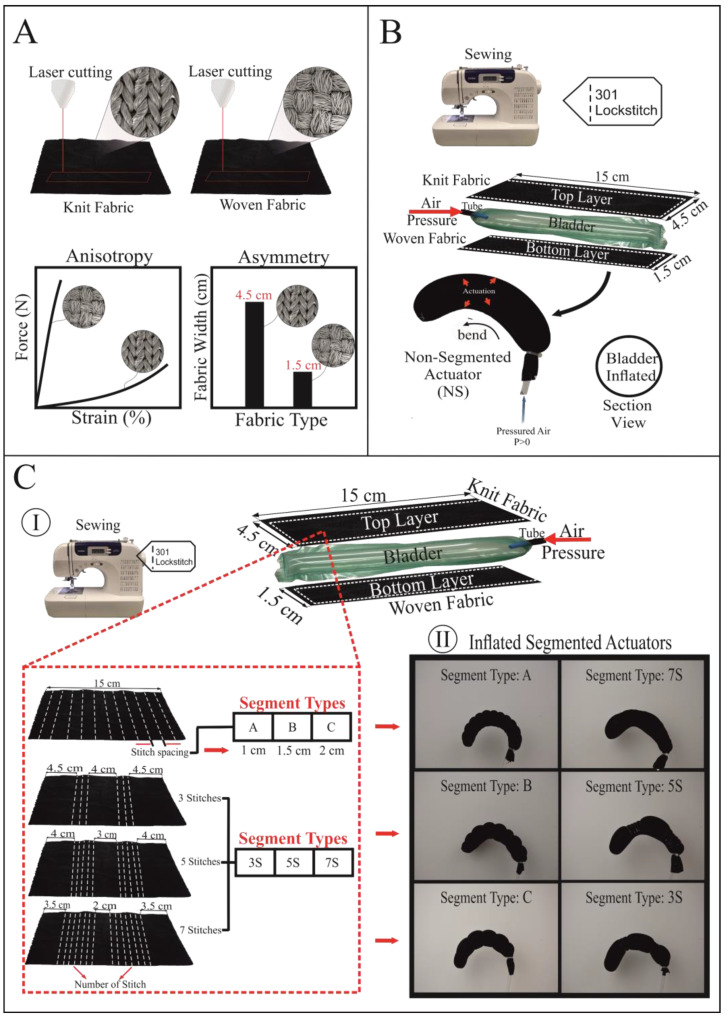
Fabrication steps of non-segmented and segmented fabric-based actuators. (**A**) Laser cutting of knit and woven fabrics, showing mechanically anisotropy and asymmetry approaches. (**B**) Construction and sewing of non-segmented actuator, demonstrating the bending motion of the inflated non-segmented actuator. (**C**) Construction of segmented actuators. (I) Sewing of segment types (A, B, C and 3S, 5S, 7S) on knit fabric. (II) Showing of pressurized states of segmented actuators.

Figure 1 depicts the fabrication method of textile actuators. The actuators are made by combining knit fabric on the top layer and woven fabric with a strain-limiting function on the bottom layer using a Brother CS 6000i sewing machine. The sewing is performed with 301 lock stitch at four tension settings. For sewing thread, 100 percent polyester Nm 60/2 thread was used. Table 3 shows that segment types, sample numbers, and fabric combinations of 28 different actuators were created. Actuators are made up of six different segment types that are A, B, C and 3S, 5S, 7S using two different knit (K1–K2) and two different woven fabrics (W1–W2). The fabric combinations in Table 3 define which woven fabric is used in the bottom layer of the actuator and which knit fabric is used in the top layer of actuator. These actuators have asymmetry due to the difference in width-wise length (woven fabric width: 1.5 cm, knitted fabric width: 4.5 cm) and anisotropy due to the inherent elasticity difference (Figure 1A). We discovered that the joining seam strength of the actuator made of woven and knitted fabric cut asymmetrically is more durable than the joining seam strength of the actuator cut symmetrically.

To make the actuator without adding segments, the woven fabric was cut into strips 15 cm long by 1.5 cm wide, and the knitted fabric was cut into strips 15 cm long by 4.5 cm wide, as shown in Figure 1A,B. The woven fabric was then reversed on the knit fabric and aligned to its right edge. On the right edge, the aligned knitted and woven fabrics were sewn with a straight stitch. After that, the same sewing process was repeated, but this time the woven fabric was aligned to the left edge of the knit fabric. After straightening the knit strip so that it was in the center of the woven strip, the upper end of the actuator was also sewn with a straight stitch, and the actuator sample was closed on three sides. Finally, the actuator’s front face was turned out. Woven and knit fabrics were cut into strips to begin the production of segmented actuators. Across the width of the knit fabric, segment stitches were made (Figure 1C). The joining process of woven fabric and segment stitched knit strip was completed in the following steps of the production of non-segmented actuators. In our study, we labeled segment types as A, B, and C, as well as 3S, 5S, and 7S. The A, B, and C segment types indicate stitch spacing between the lower and upper width-wise stitches. The joints in the human finger were taken into consideration to create the 3S, 5S, and 7S segments. The codes 3S, 5S, 7S refer to the number of stitches created separately in the upper and lower parts of the knit fabric.

### 2.3. Fabrication of Thermoplastic Air Bladder

The air bladder was manufactured using an impulse sealer machine (PCS 300, Brother, Nagoya, Japan), thermoplastic polyurethane film (Stretchlon 200, Fiber Glast, Brookville, OH, USA), and polyurethane pipe. The impulse sealer machine was set to the 5.5 s time setting in order to form a bond between two rectangular cut films that were stacked on top of one another. When all three sides of the rectangle cut TPU sheet were closed, the bonding procedure was complete. By inserting a polyurethane tube into the open end, air was forced into the bladder via a pneumatic system, inflating it. The actuators in our study were composed of a single pocket produced by two layers of fabric, and a tpu bladder of 18 cm in length and 2.8 cm in width was inserted into the actuator’s pocket.

### 2.4. Sensor Integration to Actuator

**Figure 2:**
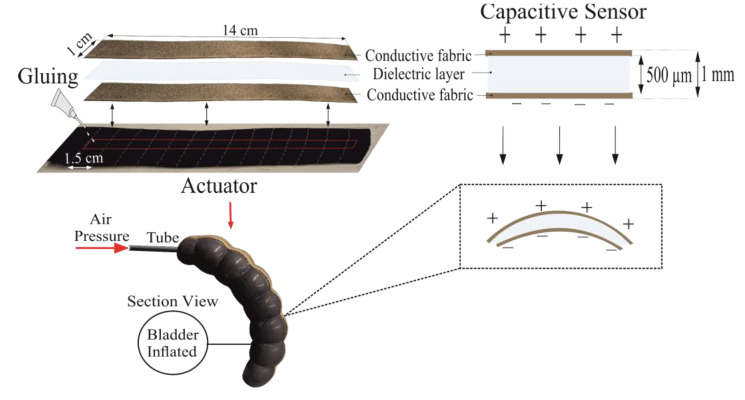
Sensor integration to actuator.

Based on our previous study, we prepared textile-based capacitive strain sensors made of conductive silver-plated knit fabric as electrodes and silicone elastomer a dielectric [31]. The capacitive sensor was bonded using flexible glue to the knit fabric, (the actuator’s upper layer) and dried, as shown in Figure 2. This procedure was performed for five actuators, which correspond to the five fingers of a wearable robotic glove. This sensor measures the elongation of the actuator during bending motion; consequently, we measured the change in capacitance and defined the corresponding bending angles of the actuators created for five fingers. The actuators were then permanently attached to the fingers of a wearable glove.

### 2.5. Tensile Test

**Figure 3:**
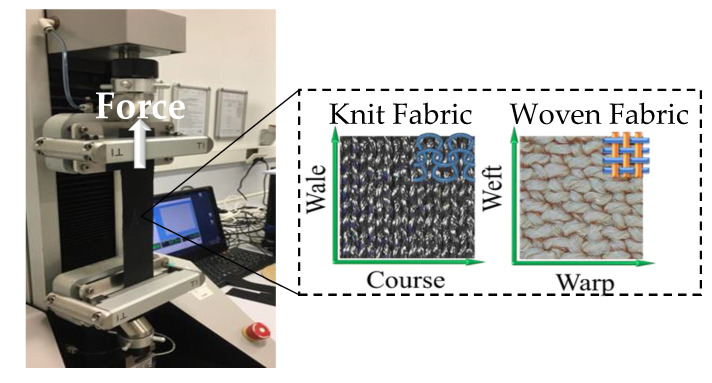
Uniaxial tensile test of knit fabric and woven fabric.

The “uniaxial test method” or the force effect method in only one direction is used to determine the anisotropic tensile properties of fabrics. In this investigation, we used the James Heal Titan Universal Testing Machine to conduct uniaxial tensile tests in accordance with the EN ISO 13934-1 test standard (Figure 3). Five strips of 20 cm × 5 cm were cut in the weft–warp directions and the course–wale directions of woven and knit fabrics, respectively. All fabrics were conditioned for 24 h prior to the test under typical atmospheric conditions. The average of five samples was used to produce the force–extension graph. The speed of the test is 100 mm/min. The goal of this test is not to measure the fabric’s strength but to determine the direction of extension and to demonstrate the differences in mechanical anisotropy between woven and knit materials. This test is critical because the bending movement of an inflated actuator is obtained when the high extension direction of the knit fabric is used in the longitudinal direction of the actuator.

### 2.6. Grip Force–Pressure Test

The soft actuators were characterized in order to understand how grasping force varies with actuator pressure using the grip force test setup demonstrated in our prior study [17]. The gripping range of each actuator was determined by the amount of pulling force applied by the actuator to the hollow rectangle attached to the dynamometer.

### 2.7. Bending Angle–Pressure Test

To better understand the bending behavior of the single chamber soft actuator, the change in its bending angle in relation to the applied pressure is recorded for each actuator as it is gradually inflated. To provide consistency between actuator samples and to facilitate understanding, bending angle is widely utilized in comparison with the curvature value [16] when measuring the actuators’ bending motion.

To eliminate the variation in brightness caused by the lighting conditions in the room, the actuator was placed in a closed platform that included a camera, an installation fixture, and a single-channel test rig. An air pump, a vacuum pump, an inflate valve, a deflate valve, a vacuum valve, a 4-channel relay board, an air pressure sensor, an MPR121 capacitive touch sensor controller, manual switches, and a power supply comprise the single-channel test setup [17]. Using manual switches, the test rig can inflate, deflate, or vacuum the actuator’s bladder. Due to the nature of TPUs, vacuum is the state of full suction of the air inside the bladder, which cannot be obtained when utilizing the deflate command. The test equipment can monitor and communicate bladder air pressure and actuator capacitive sensor values to the computer. During the tests, the proximal end of the actuator was secured to the installation fixture to eliminate any unwanted motion, while the distal end moved freely in a circular motion.

A digital camera (Microsoft LifeCam HD-3000, Microsoft, Beijing, China) positioned perpendicular to the actuator’s platform captured the bending action of the actuator. Three color markers were attached to the neural axis of the actuator, and their location was fixed in accordance with the finger knuckles to establish the bending angle. As a result, the extreme points are the bottom of the proximal phalanx (M1), the middle of the medial phalanx (M2), and the top of the distal phalanx (M3).

By using automated contour detection and circle-fitting algorithms operating in real time, the coordinates of each marker’s centroid in each frame of the captured video are identified. Thus, the angle formed by M1, M2, and M3, namely angle *A*, is calculated by the Cosine formula:

(1) $$A=\cos^{-1}\left(\frac{b^{2}+c^{2}-a^{2}}{2\mathrm{bc}}\right)$$

where *b* is the distance between M1 and M2, *c* is the distance between M2 and M3, and *a* is the distance between M1 and M3. The bending angle is calculated as the conjugate angle to the central angle that sub-tends to the inscribed angle, *A*.

To demonstrate the experiment’s repeatability, the actuator’s inflation and deflation processes were repeated six times sequentially. Due to the fact that the number of frames collected per second and the pressure values obtained were varied, these values were matched using their timestamps. To produce more stable results, the initial cycle of pressure application and withdrawal was omitted. After obtaining the matched data, just the inflation component was retrieved, as only the inflation component contained valuable information on the bending motion. The average pressure value corresponding to each bending angle was calculated. This procedure was followed for all actuator types.

### 2.8. Torque–Pressure Test

To determine the twisting force exerted by the actuator around its own proximal end, the value of the exerted torque was recorded at various bending angle configurations. To replicate the range of finger motion, the bending angle was set between 0° and 75° in multiples of 15°. The actuator’s distal end was inserted into a hollow cylinder created using a three-dimensional (3D) printer and affixed to the top of the dynamometer to apply a pulling force during the experiments. The distal end was fastened in a solid 3D-printed object and relocated for the purpose of collecting torque values at various bending angle configurations. During the torque experiment, the actuator was inflated and deflated four times sequentially with the same static pressure speed, and the related statistical variables were calculated. The torque test was conducted in the same manner as the grip force test in terms of data collecting and post-processing. To determine the torque value, the actuator with the best grip force and bending angle was chosen and included in the experiment setup. To calculate the net output torque, *τ*, the product of the dynamometer pressure value (*F*) and the actuator–dynamometer distance in mm (*r*) was used.

*τ* = *r* · *F* (2)

### 2.9. Radial Expansion Test

**Figure 4:**
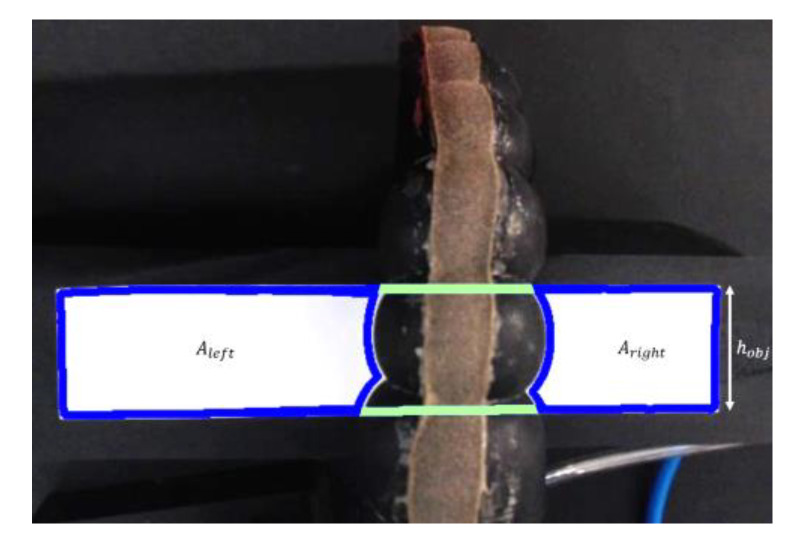
Camera view of the radial expansion test setup.

In unrestricted designs, radial expansion of the actuator walls results in an increase in its diameter and a decrease in bending angle over a wide range of pressure levels. The radial expansion of two distinct actuators is determined, and their performance is compared in relation to the applied internal pressure. To accomplish this, the actuator was put in an isolated area that was internally illuminated in order to maintain a static lightning state during the experiment. A rectangular object with a defined width and height and a different color than the actuator was positioned beneath the actuator, and the camera installed at the top of the experiment environment captured the actuator’s motion over the object. Due of the color-based method used, the experiment area was intended to contrast with the object. The outside contours of the viewable parts of the item were generated using image processing techniques. As observed in Figure 4, the object looked to be divided into three sections from the camera as a result of the actuator being placed on it. The regions covered by the right and left sections can be identified based on the object’s color. However, the region of the middle part cannot be detected because it is beneath the actuator. Given the shape of the object, the region it covers may be determined by adding the upper and lower lines of the right and left sections.

The number of pixels in the region occupied by the object indicates the area of the object in the image. Using the formula in Equation (3), the pixel per square cm (*PPcm*^2^) value in the image is found using:

(3) $$PPcm^{2}=\frac{A_{obj}}{{\hat{A}}_{obj}}$$

where *A_obj_* is area of the object in cm^2^ and *Â_obj_* is the number of pixels in the region occupied by the object. In other words, *Â_obj_* is the pixel area of the object in the image. The region underneath the actuator provides information about the actuator diameter. The pixel area of the region underneath the actuator is calculated as:

(4) $${\hat{A}}_{under}={\hat{A}}_{obj}-{\hat{A}}_{right}-{\hat{A}}_{left}$$

where *A_hidden_* is the pixel area of the region hidden by the actuator, *A_right_* is the pixel area of the right part of the object, and *A_left_* is the pixel area of the left part of the object. Then, real area of the region underneath the actuator can be calculated using *PPcm*^2^ and *A_hidden_* as below:

(5) $$A_{under}=A_{hidden}\cdot PPcm^{2}$$

Furthermore, the actual area of the region hidden by the actuator can be calculated as below:

(6) $$A_{under}={\bar{d}}_{act}\cdot h_{obj}$$

where $\bar{d}$*_act_* is the average diameter of the actuator, and *h_obj_* is height of the object. Using both formulas, the average diameter of the actuator can be calculated as below:

(7) $${\bar{d}}_{act}=\frac{{\hat{A}}_{under}\cdot PPcm^{2}}{h_{obj}}$$

Actuator radial expansion and the air pressure data are collected simultaneously in a real-time manner.

### 2.10. Capacitance Measurement

The constant DC charge approach was utilized in this work to measure capacitive sensors. This technique charges the sensor with a constant current value over a constant time period. Thus, capacitance stores a charge, which may be calculated using Equation (8):

(8) Q=I·t

where *Q* is charge amount, *I* is the current of the charge, and *t* is the duration of the charge. After the charging process, the voltage information of the sensor can be measured, and the capacitance value can be calculated using Equation (9):

(9) Q=C·V

where *C* is the capacitance value of the sensor, and *V* is the voltage value of the sensor after the charging. Using both equations, the capacitance value is calculated as follows:

(10) $$C=\frac{I\cdot t}{V}$$

It is possible to increase the sensitivity of capacitance measurement by changing the current and charging time parameters in this technique.

Capacitance is measured using the MPR121: proximity capacitive touch sensor controller. The integrated circuit’s primary role is to detect the body capacitance using the electrodes produced for the button and keypad. The capacitance of the human body fluctuates between 50 pF and 250 pF [32], and capacitive sensors produced with textiles do not surpass this value. As a result, this integration can also be used in capacitive sensors based on textiles. Charge current and time values can be modified in software on this integrated circuit, allowing for more precise measurement of capacitances across a wide range. Additionally, the integrated circuit’s two-layer digital filter reduces noise during capacitance measurements. This integrated circuit is capable of measuring up to 12 electrodes/sensors [33].

### 2.11. Control Strategy

**Figure 5:**
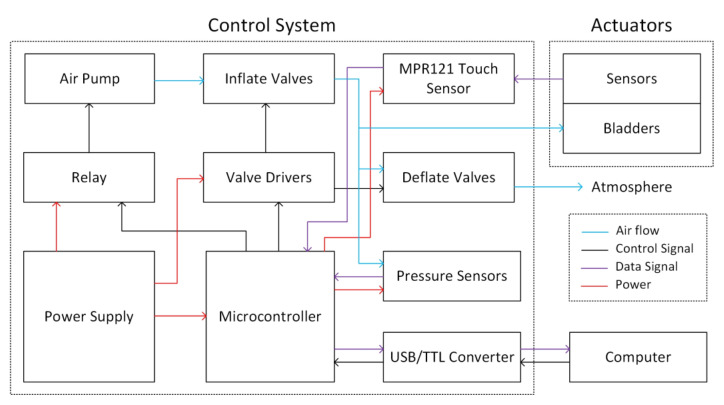
Control system of sensorized soft robotic glove.

A control system is prepared for managing the operation of the prototype glove mentioned in Section 3.3. Figure 5 shows the block diagram of the developed control system. The control system consists of air flow components, control components, data components, and power components.

Components of airflow include an air pump, inflate valves, deflate valves, and pressure sensors. To initiate the actuators’ bending motion, the air pump is activated, and the inflate valve is opened. The generated air is directed to the bladders via the inflate valves, and the actuator is bent. While the actuator is bending, the deflate valve is in the closed position. It is worth noting that no two air bladders or actuators are completely identical due to manufacturing conditions. When non-identical air bladders were filled simultaneously, a homogeneous bending action in all fingers was not observed. As a result, the actuators are inflated sequentially with a 200 ms inflation duration apiece, while the entire glove forms the closing movement. If all actuators meet the stop condition, no additional air is provided to the actuator that meets the stop condition, and the air pump is shut off. The air pump and inflate valves remain closed during the relax movement of the glove, while the deflate valves are simultaneously opened. Thus, the air in the air bladder flows toward the atmosphere because its pressure exceeds that of the surrounding air. When the glove provides a stopping condition for the sensors-measured relax motion, the deflate valves are closed. 

The control system consists of the following components: a relay, valve drivers, a microcontroller, a USB/TTL converter, and a computer. The microcontroller controls the actuator by receiving opening and closing signals from the computer. Additionally, it controls whether the actuator is closed or opened in response to sensor input. A USB/TTL converter connects the computer and the microcontroller. When the air pump is functioning, the microcontroller may control it through the relay, and it operates at full power. Valve drivers are used to regulate valves, as a heating problem develops when the valves are left open with full power for an extended period of time; thus, the valves are opened with maximum power for 10 milliseconds and then operated with low energy to maintain the open state.

**Figure 6:**
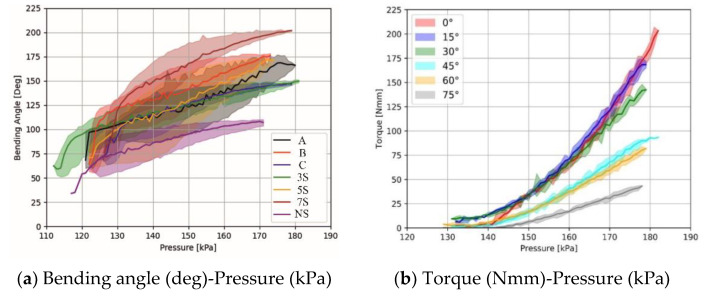

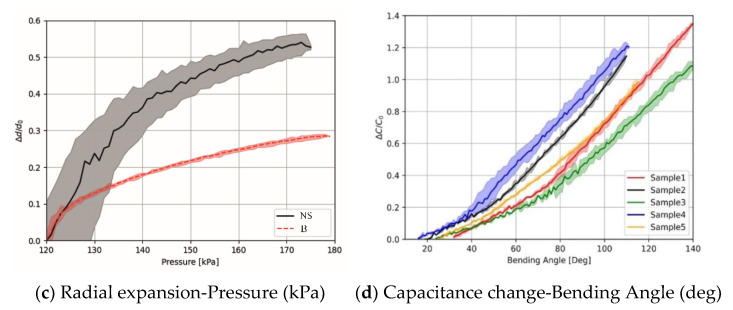
The experimental results of actuators.

The MPR121 touch sensor, pressure sensor, microcontroller, USB/TTL converter, and computer are the signal components. When capacitive sensors are used, the noise in the sensor values increases as the distance between the measurement circuit, and the capacitive sensor grows due to changes in the line capacitance. Thus, noise can be minimized by positioning the measuring circuit as close as feasible to the sensors [34]. To minimize the noise, the MPR121 touch sensor was placed closest to the actuators. Capacitive sensors generate feedback signals, while the actuators carry out the given commands, and the system is controlled by a closed-loop control system. According to the results in Figure 6d, the capacitive sensors show approximately linear behavior with respect to the bending angle. In this way, with the upper and lower threshold values determined for the sensor values, it can be decided whether the actuator is in the closed or open state. By scaling the upper and lower thresholds, the actuator can be closed at different angles. According to the results in Figure 6d, capacitive sensors on 5 actuators show similar behavior but operate with different sensitivity. For this reason, when the system is started, all actuators are calibrated one by one, and separate upper and lower threshold values are determined for each. These obtained upper and lower limits constitute the bending and relax stop conditions of the actuators. When an actuator reaches its upper limit in inflation, it meets the stop condition and is no longer supplied with airflow. Similarly, when the capacitive sensors reach the lower limit in the relax state, the deflate valves are closed. Pressure sensors are used to control the air pressures in the actuators and provide a backup feedback signal in case any problem occurs with the capacitive sensors. Both capacitance and pressure sensor values are collected by the microcontroller, and control signals are generated according to these sensors’ data. At the same time, the sensor data are transferred to the computer via USB/TTL converter and used to visualize the system data.

The system is powered by a 12V 16A power supply. This power supply also feeds the microcontroller, relays, and valve drivers. The pressure sensor and MPR121 are fed with appropriate voltages through the voltage regulator connected to the microcontroller. Due to the voltage difference between the microcontroller and the power supply, it is given to the microcontroller after the voltage is reduced to the appropriate level with the adjustable voltage regulator.

## 3. Results and Discussion

### 3.1. Uniaxial Tensile Characteristic of Woven and Knit Fabrics

When inflated with air, fabric actuators elongate and deform in various directions. The anisotropic behavior generated by the variation in elongation of the textiles is employed by the actuator to perform the bending movement. In this study, to determine how anisotropy difference of textile materials affects actuator performance, a comparison of the tensile characteristics of the woven and knit fabrics that make up the actuator structure was made.

**Figure 7:**
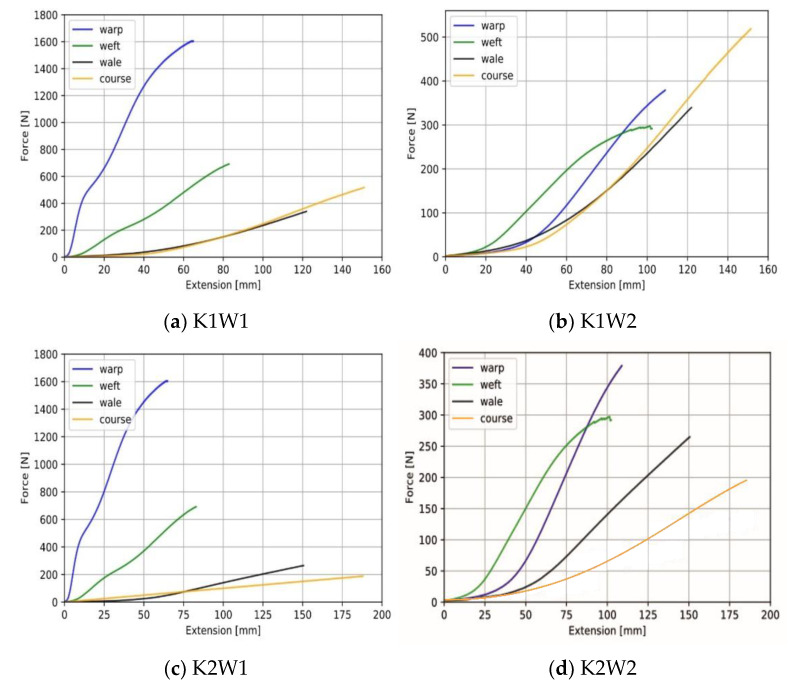
Results of uniaxial tensile test of textile fabrics.

The results of the force–extension test of combinations of knit fabrics and woven fabrics are given in Figure 7. The extension until failure for woven 1 (W1) fabric in the warp direction was 65 mm at 1600 N, and in weft direction, 83 mm at 700 N; for woven 2 (W2) fabric in warp direction was 110 mm at 390 N, and in weft direction, 100 mm at 300 N. The extension until failure for knit 1 (K1) fabric in the course direction was 150 mm at 530 N, and in wale direction, 125 mm at 340 N; for knit 2 (K2) fabric in course direction was 188 mm at 198 N, and in wale direction, 150 mm at 260 N. According to the results of the uniaxial tensile test of textile fabrics, an anisotropy comparison was made in two main directions, warp–weft for woven fabric and course–wale for knit fabric. Knit and woven fabrics exhibited different deformations as a result of fiber content, linear density, fabric geometry, yarn properties, and related Poisson effects in response to applied loading. Knit fabrics showed more deformation than woven fabrics due to their loop structures and elastane yarn content. Due to the elongation of the course direction of knit fabrics and the warp direction of woven fabrics, the longitudinal placement of the actuator was made in these directions. The woven 1 fabric was more rigid than the woven 2 fabric due to its coated structure, that is, it showed a low elongation tendency when high force was applied. In addition, it was concluded that the anisotropy difference between the warp direction of the woven 1 fabric and knit fabrics (K1 and K2) was very high. Among the fabric combinations, K1W1 and K2W1 had the highest anisotropy difference between woven and knit layers, and the lowest anisotropy difference was noted for K1W2.

### 3.2. Characterization of Fabric-Based Actuators

**Figure 8:**
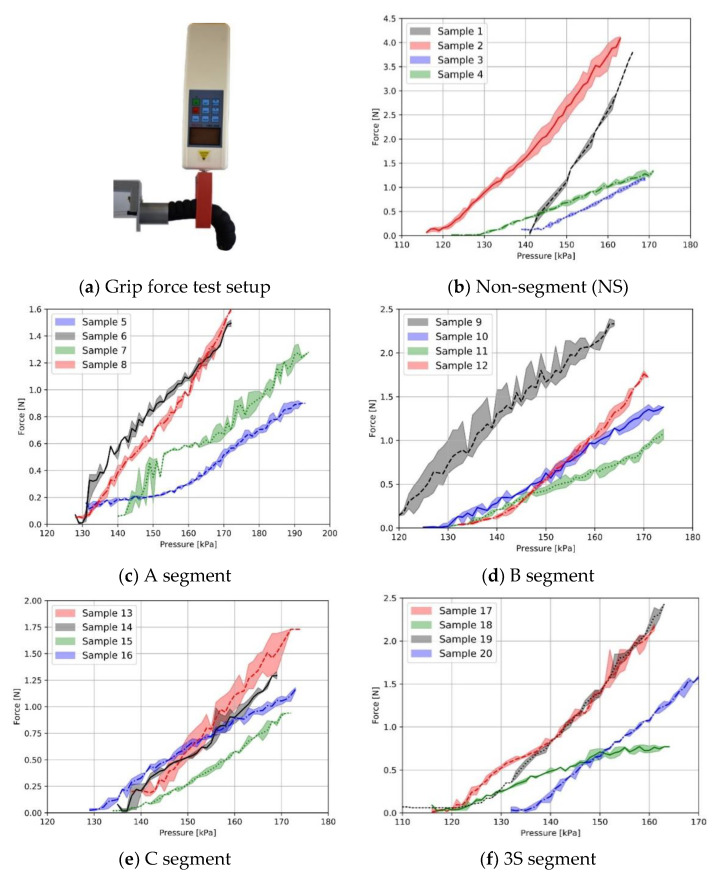

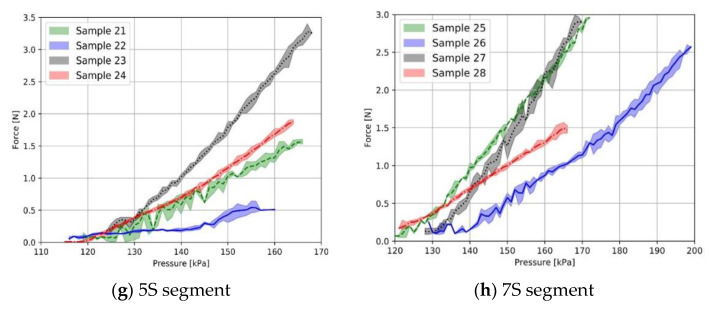
Grip force–pressure test.

The effect of segment types on the gripping force of the actuator was investigated. The graphs of the applied pressure versus the grip force of the segmented and non-segment actuators in all fabric combinations are given in the Figure 8. The non-segmented sample 2 (K2W1) had the maximum gripping force compared to all actuators (4 N at 160 kPa). Sample 1 (K1W1) and sample 2 (K2W1) combinations, which showed the highest anisotropy difference, achieved the highest grip force. It has been found that actuators with K1W1 fabric combination generally produce high gripping force with low pressure change. Forming A, B, C and 3S, 5S, 7S segment types decreased the grip force in K1W1 and K2W1 samples that have a high anisotropy difference. Creating different segment types in samples with a low anisotropy difference resulted in unstable behavior on grip force. This is because the uncoated woven 2 fabric structure can affect the complicated nature of the textile’s strain deformation.

The bending angles of all segmented and non-segmented actuators in the K1W1 fabric combination against pressure are given in Figure 6a. Segmented and non-segmented actuators exhibited bending behavior; however, a higher bending angle was obtained with segmented samples. At 170 kPa, the non-segmented actuator had a maximum 110 degree bending angle. It was noticed that inflating the actuator to a pressure greater than this value had no effect on the bending angle. All segmented actuators can withstand pressures more than 170 kPa and have bending angles between 150 and 200 degrees. As a result, it was determined that increasing the number of segments increased the bending angle. It is believed that creating stitches in the width-wise direction on the actuator’s knit fabric increases the bending angle by preventing the actuator from expanding radially. The actuator with the greatest bending angle was determined to be a 7S segment type K1W1 specimen. However, due to the radial expansion irregularity between the non-segmented parts without stitching and the segmented parts with stitching, it was determined that this actuator would not be suitable for using in the application of wearable gloves. As a result, the B segment type (1.5 cm stitch gaps) K1W1 actuator was chosen, as it had the second highest bending angle.

The experimental results of the torque measurement are shown in Figure 6b. The reported results show that the B segmented approach produces the highest torque with a maximum of 210 N.mm at 0 degrees bending when a pressure of 183 kPa is applied. It was found that as the bending increased, the torque produced by the fabric actuator decreased. This result is consistent with the torque results in our previous work [17].

The radial change experimental results are shown in Figure 6c. Fabric actuators initially are the two-dimensional (2D) and expand radially with a given pressure increase. The non-segmented actuator inflates as a cylindrical tube, while the segmented actuator exhibits a knobby appearance. A radial change of 0.28 mm of the segmented actuator was achieved at the maximum pressure of 175 kPa, while the non-segmented approach achieved a radial change of 0.53 mm at the same maximum pressure. It was observed that the non-segmented actuator had high radial expansion due to the tendency of the weft-knitted fabric to elongate in the width-wise and longitudinal directions. It is not aesthetically pleasing to place an actuator that highly inflates in the radial direction as an exoskeleton on top of the fingers. In addition, compressed air contributes to the inflating of the actuator, not its bending. When comparing the segmented actuator created by adding 1.5 cm stitch gaps (B) and the non-segmented actuator, it was found that the radial expansion was reduced by approximately 50%.

Figure 6d shows capacitance change–bending angle graph of five identical actuators with integrated textile-based capacitive strain sensors. These actuators have B segment structure and K1W1 fabric combination, which depicts the best performance according to bending angle and grip force. As can be seen from the results, the capacitance change and bending angle show positive correlation. The sample results are approximately linear. Although the sensitivity of identically produced actuators is different according to the bending angle, they present similar behavior.

### 3.3. Application

**Figure 9:**
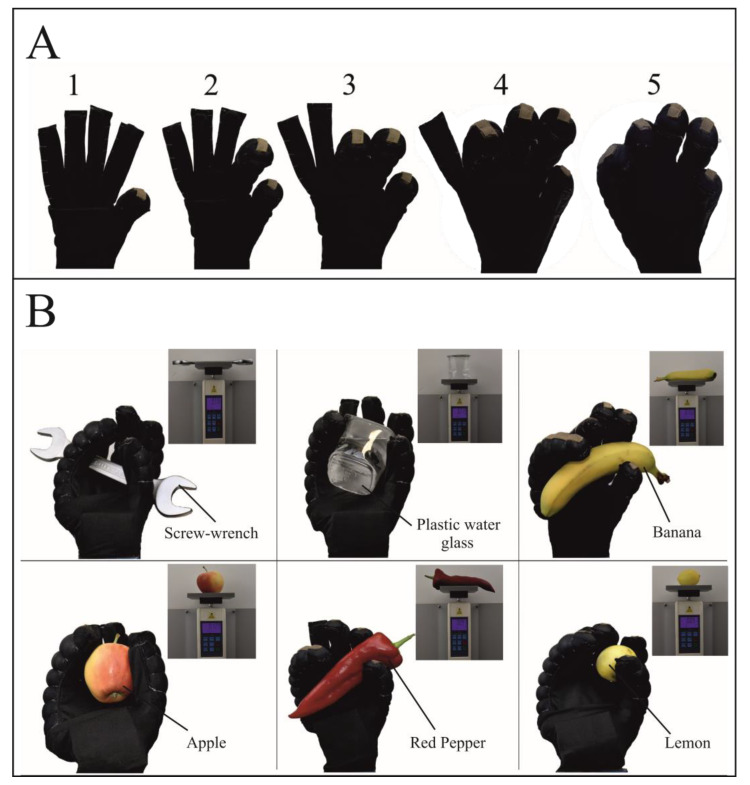
Application of soft robotic glove. (**A**) Demonstrating how each of the five fingers on the robotic glove can be controlled separately. (**B**) Holding several objects of various shapes and weights by soft robotic glove.

To demonstrate the application of the sensorized fabric-based pneumatic actuator, it was integrated into a wearable glove. The glove was made of lycra/polyester fabric and was stretchable and compliant. To demonstrate the glove’s actual holding capability without human intervention, the glove was worn by a hand model. As shown in Figure 9A, all fingers were respectively given a closing motion and represented by the numbers 1-2-3-4-5 to prove the separate controllability of the actuators that are integrated into all five fingers of the glove. By operating the control system of the developed soft robotic glove, the flexion movement of the actuators was enabled to hold various objects at 160–180 kPa. Actuator fingers were inflated to create different grasp types according to the shape of the objects. As seen in Figure 9B, the objects that can be found in daily life were selected to be different sizes and weights, including: screw-wrench (336 g), plastic water glass (219 g), banana (150 g), apple (129 g), red pepper (93 g) and lemon (51 g). Some objects had a slippery surface, causing then to be difficult to hold. Therefore, it is recommended to develop approaches that will create friction between the glove and the object.

## 4. Conclusions

The stitching technique was used to create segmented fabric-based bending actuators in this study. The influence of six distinct segment types was tested on the actuators’ grip force and bending angle. Four different combinations of knit-woven materials with varying degrees of mechanical anisotropy were selected to produce actuators. It was discovered that actuators with a large anisotropy difference have a greater grip force than actuators with a small anisotropy difference. Segmentation by adding width-wise stitches reduces the grip force in samples with a large anisotropy difference. By adding segments, the actuator’s bending angle is increased, while actuator’s radial expansion tendency is decreased. It is also recommended to employ coated woven fabric in actuators made of knit-woven fabric to achieve repeatable results. The capacitive sensors demonstrated the controllability of the soft robotic glove using a closed-loop system. The developed sensing soft robotic glove can provide sufficient force for objects weighing up to 336 g and of different shapes due to the wide range of bending angles and grip forces achieved. This demonstrates the wearability and application of the glove as an exoskeleton device for people who want to perform daily life activities.

## Figures and Tables

**Table 1:** Properties of knitted fabric samples.

Properties of Fabrics	Knit Fabric 1 (K1)	Knit Fabric 2 (K2)
GSM (g/m^2^)	180	235
Course per cm	41	50
Wale per cm	35	26
Thickness (mm)	0.58	0.48
Fiber Type	Polyester/Elastane	Polyamide/Elastane
Knit Structure	Single Jersey	Single Jersey

**Table 2:** Properties of woven fabric samples.

Properties of Fabrics	Woven Fabric 1 (W1)	Woven Fabric 2 (W2)
GSM (g/m^2^)	195	165
Warp density (ends/cm)	52	34
Weft density (picks/cm)	35	24
Thickness (mm)	0.45	0.41
Fiber Type	Polyester	Polyester
Weave Structure	Coated Plain Weave	Plain Weave

**Table 3:** Segment types and fabric combinations of actuator samples.

Segment Types	Sample	Fabric Combinations
Non-Segmented (NS)	1	K1W1
	2	K2W1
	3	K1W2
	4	K2W2
A	5	K1W2
	6	K1W1
	7	K2W2
	8	K2W1
B	9	K1W1
	10	K1W2
	11	K2W2
	12	K2W1
C	13	K2W1
	14	K1W1
	15	K2W2
	16	K1W2
3S	17	K2W1
	18	K2W2
	19	K1W1
	20	K1W2
5S	21	K2W2
	22	K1W2
	23	K1W1
	24	K2W1
7S	25	K2W2
	26	K1W2
	27	K1W1
	28	K2W1

## Data Availability

The data presented in this study are available on request from the corresponding author.
